# Social capital and mental health among mothers in Vietnam who have children with disabilities

**DOI:** 10.3402/gha.v6i0.18886

**Published:** 2013-02-11

**Authors:** Nguyen Thi Minh Thuy, Helen L. Berry

**Affiliations:** 1 Department of Community Based Rehabilitation, Hanoi School of Public Health, Hanoi, Vietnam; 2 Faculty of Health, The University of Canberra, Australia

**Keywords:** distress, social capital, mothers, children with disabilities, community participation, personal social cohesion, women's health, mental health

## Abstract

**Background:**

Having a child with a disability is a heavy burden for mothers, especially in developing countries, where there is little available financial or other government support. Having a child with a disability is also linked to mental health problems and poor quality of life. Communities rich in social capital and individuals who have high levels of personal social capital generally enjoy day-to-day and long-term health and social benefits but this has not been investigated in Vietnam among mothers of children with disabilities. This study aims to investigate these mothers’ distress in terms of their social capital.

**Methods:**

A cross-sectional study based on an interviewer-assisted survey included 172 mothers of children with moderate/severe disabilities in two provinces of Vietnam (one in the North and one in central Vietnam), using a newly translated and modified version of the Australian community participation questionnaire, several measures of personal social cohesion, and Kessler's 10-item measure of general psychological distress. Hierarchical linear regression modelling was used to explore the relationships among socio-demographic factors, multiple components of structural and cognitive social capita, and mothers’ distress controlling for a wide range of socio-demographic characteristics, the nature of the child's disability, and mothers’ personality (extroversion).

**Results:**

Mothers in this study were highly and multiply disadvantaged, and they had very high levels of distress and low levels of community participation. Furthermore, most forms of participation were associated with greater, not less, distress. Socio-demographic characteristics, child's disability, and mothers’ personality did little to explain variance in mothers’ distress, but types and amounts of participation were important predictors. The final regression model explained 29% of variance in distress, with major contributions made by living in a mountainous area, having a ‘reserved’ personality, and frequency and types of participation.

**Conclusion:**

Vietnamese mothers whose children have disabilities are extremely marginalised and distressed. They have only modest social capital, but the little they have tends to be related to better mental health. Being from the mountains; being ‘reserved’; spending time with friends, neighbours, and in educational activities; and trusting others are related to better mental health among these women. However, several types of participation are associated with worse mental health. Such activities should be avoided in any interventions designed to increase social capital as a mental health promotion strategy.

Family members’ expectations, hopes, and plans, including work and financial arrangements, rest on the expectation of having a ‘normal’ child, not a child with disabilities. The birth of a child with a disability has adverse consequences for the lives, emotions, and behaviours of all family members. These consequences arise because looking after a child with a disability is very difficult and families are not prepared for it (1). Families with children with disabilities experience very high levels of stress and depression (2–4). Throughout their lives, parents of these children report a wide array of difficult emotions: protectiveness towards someone helpless; revulsion at the abnormal; reproductive inadequacy; child-rearing inadequacy; anger; grief; shock; guilt; and embarrassment (5). The high prevalence of psychological distress these parents endure stems from the interplay of multiple factors. These include child factors, such as age, sex, type of disability, caregiving ‘burden’, the presence of externalising behaviour problems, and emotional disorders. Parental factors, such as personality and coping styles, and social factors, such as marital harmony, social support, and socio-economic circumstances, also play an important part (4).

**Policy Recommendations**

Results of the study suggested the following recommendations:

- Vietnamese women with children with disabilities have very poor mental health. Where possible, mental health intervention services should be made freely available to these women.
- These women also have very limited social contact. Providing a setting in which they could interact socially could help meet their need for social contact and friendship in a non-threatening environment through being among women in similar circumstances. The social contact, support, and opportunity to develop closeness and trust could help buffer their mental health.
- Educational participation was associated with better mental health among Vietnamese women with children with disabilities. Educational opportunities specially designed for these women could provide educational opportunities as well as offer social contact.
- Poverty is a real problem among Vietnamese women with children with disabilities. These women, their children and their whole families need financial support to relieve some of the pressure they experience daily. It could also mean that they need to work a little less hard, freeing up a small proportion of their time for mental health-promoting social and educational contact.

## The impact of children with disabilities on parental wellbeing

The birth of a normal or abnormal child is viewed as the mother's personal success or failure, respectively. When a child is born with a disability, the mother is often blamed and belittled by society, and false explanations may be attributed to her child's disability, leading to her feeling hopeless about the future (1). Mothers, more so than fathers, are at higher risk of depression because they tend to take active roles in caring for their children with disabilities, even relinquishing their jobs or leisure activities (6). They also report significantly greater anxiety than other mothers (2) and worry about their children's future and ability to find a place in society. Depression, these difficult emotions, and the many hardships they face in addressing their children's problems are associated with low levels of energy and physical activity, affecting their general quality of life (3). Compared with parents of healthy children, parents (especially mothers) of children with disabilities have reported impairment in physical activity, health and social relationships, as well as worse overall perceptions of their quality of life (7).

## Family influences on the development of children with disabilities

In addition to child impacts on parents’ wellbeing, family factors, especially mothers’ circumstances, affect the children's development (8). Whitely (9) explored the relationship between family stressors and dysfunctionality and psychosocial adjustment in children with disabilities. Family factors were more predictive of children's psychosocial adjustment than their disabilities. The strain on families, poor maternal physical or mental health, and poverty were the strongest correlates of maladjustment in these children. Mothers’ mental health is particularly important. Compared with other children with disabilities, those with depressed mothers show more behavioural disturbance, poorer cognitive functioning, more insecure attachment, a more difficult temperament, and greater risk for developing depression when older (10).

Despite the evident need for assistance for families with children with disabilities, providing supplementary financial support for these families may not, ultimately, much improve their health and wellbeing. In the United Kingdom, ‘Family Fund’ grants were given to families of children with disabilities, providing them with washing machines, tumble dryers, refrigerators, and telephones. In addition, financial grants were made to purchase items, such as bedding and clothing, or holidays and day outings. The evaluation of these grants showed that the support did not improve the extent to which the disability adversely affected families’ lives (11). Nevertheless, mothers reported a greater sense of wellbeing and fewer symptoms indicative of mental and physical ill-health. The evaluation concluded that it was essential to provide an integrated package of services to help parents cope effectively with the many difficulties associated with caring for their children.

## Social capital in the lives of mothers with children with disabilities

To understand how living with children with disabilities affects family wellbeing, it is important to consider the family's broad social context (12). Much of the time that might be available to caregivers for social participation is consumed by caring responsibilities. With the arrival of a child with disabilities, family networks and support often diminish quickly (13). More significantly, people with disabilities and their families endure substantial stigma and social exclusion, leaving them unable to participate in society or to access services equitably (13). Some families choose not to attend community events to avoid confronting the stigma or their own difficult feelings towards those who exclude them (13). These dynamics can lead to a dramatic deficit in social capital for these families.

Social capital comprises two components, community participation and social cohesion (23). A deficit in social capital matters because social capital is widely considered a critical element of public health promotion and a reliable predictor of health, happiness, and life satisfaction (14). It is made up of two separate but connected components: community participation and personal social cohesion (15), also respectively known as the structural and cognitive components of social capital (16–18) or what people ‘do’ and what they ‘feel’ (19). The structural component has to do with multiple ways of participating in the community, the networks of association that participation generates, and the quality of relationships (18) within and between those networks. Participation is thought to be linked to cohesion in that greater participation leads to greater cohesion (20), creating a virtuous circle of social capital creation and maintenance. Social capital can be measured at various group levels (such as at neighbourhood or provincial level) or at the individual level. In the latter case, this may be termed ‘personal social capital’ (21). It comprises individuals’ patterns of community participation and their ‘personal social cohesion’ (20, 22). Because personal social capital is strongly protectively related to health, especially to mental health (23), loss of social capital is a substantial concern for these at-risk families.

The stigma and exclusion that follows having a child with disabilities harm these families because they inhibit participation (24), breaking the positive cycle of social capital creation and maintenance. A study of people with disabilities in New York found that deficits in social capital, especially fewer social ties, were more influential than disability or economic circumstances in predicting homelessness (25). A recent review of mothers with children with disabilities reported similar conclusions, finding that participation and social support mediated and predicted health and quality of life (26). The review found that (i) greater social network size, activity, and satisfaction with network were related to greater wellbeing, particularly to life satisfaction and health; (ii) caregiver depression was significantly related to perceived inadequacy of social support; and (iii) social support was protective against declines in physical health, those caregivers who reported higher initial levels of social support showed *improved* health over time. Indeed, mothers of children with disabilities who participated in support groups often generated new social capital by establishing informal support systems for themselves and their families. They were assertive and expressive, and fairly confident that their actions could make a difference in their children's lives. Non-participating mothers were more depressed, confused, isolated, and overwhelmed. They thought of their children's disabilities as random events or genetic accidents, and could not find meaning in, or make sense of their circumstances (27).

## Children with disabilities in Vietnam

Over the past decade, numerous estimates have been made of the prevalence of disability among children in Vietnam. Estimates indicate that approximately 2–6% of the child population have disabilities (28). According to the 1999 national survey, just over 3% of Vietnamese children aged 0–17 had disabilities, nearly 1 million such children, and their situation was poor (29). Almost 50% of school-aged children (6–17 years) with disabilities were illiterate, over one-third had never attended school, and another one-sixth had dropped out of school. About one-third of these children's families had never sought treatment for their disability. Only 5% in urban areas and 10% in rural areas received any form of financial support from the government or the community, such as monthly allowances, free or subsidised education, or free health care cards. This additional disadvantage adds to pressures on these families, especially on mothers, who work very hard in Vietnam (30). Little is known about the social and health circumstances of these mothers, despite their obvious hardship and consequent need for support.

## Aims of the study

Participating in the community and a sense of cohesion, of feeling connected to the community (core components of social capital), so influence the lives of mothers of children with disabilities that interventions to improve their quality of life must include elements that enhance their social capital. However, there has not yet been any research into the relationship between social capital, health, and wellbeing in Vietnam among mothers with children with disabilities. Our study aims to (i) explore the relationship between mental health and social capital in this vulnerable group of mothers in Vietnam and (ii) consider whether and, if so, how, social capital might be promoted to enhance their wellbeing.

## Method

### Respondents and procedure

Respondents included 172 mothers aged 25–65 years (M=40.22 years, SD=8.13, Md=38 years) of children with moderate to severe disabilities living in two provinces in Vietnam, Ninh Binh (in the north) and Quang Nam (central Vietnam). The families came from four districts within these provinces, one remote mountainous, one coastal, and two rural delta districts. The women were identified from a list of children with moderate and severe disabilities provided by Catholic Relief Services, an American NGO, which supports the inclusion of children with disabilities in mainstream community schools. The children's disabilities were screened, initially, through a community-based survey and then confirmed by specialist doctors. There were approximately 220 children with moderate and severe disabilities in the project provinces, and all of their mothers were invited to participate in the study via interviewer-assisted questionnaire. Of the 175 women that attended, three declined to take part, leaving 172, a response rate of approximately 80%.

### Procedure

As this is the first time, the measures described below have been used in survey-based epidemiological research in Vietnam, and as the questionnaire was developed in English, we undertook extensive piloting. Two assistant lecturers from the Rehabilitation Department of the Hanoi School of Population Health assisted in translating and piloting the questionnaire, and collected the data. We translated the questionnaire into Vietnamese in July 2007, piloting it the following month with 11 mothers whose children with disabilities were receiving rehabilitation services at the National Institute of Paediatrics in Hanoi. We included the full 63-item version of the Australian community participation questionnaire (‘ACPQ’, see below) on the same persons on different days to identify problems with the questionnaires and its test–retest reliability. The mothers found this tiring because the questionnaire was too long and there were problems with terminology: some of the words, as we had translated them, and contexts were not appropriate in Vietnamese (for example, some items referred to attending ‘church’, though almost all Vietnamese people are Buddhist). Some concepts in the questionnaire were new, too, such as ‘self-efficacy’. Also, mothers of children with disabilities are typically poorly educated and not familiar with completing surveys, making the questionnaire much longer to complete.

We piloted a second, revised and shortened, questionnaire, which included a 30-item short-form of the ACPQ, with 88 in-service students at the Hanoi school of Public Health. The students were asked to complete the questions and to provide written comments on the questionnaire itself. The second questionnaire was more easily answered, but one-quarter of the students commented that some items still did not make sense. For example, items about ‘signing petitions’ or volunteering for ‘non-profit organisations’ were not meaningful in Vietnam where such activities are uncommon. A third, further revised questionnaire was piloted among five Catholic Relief Services staff with proficient English who were experienced in working with families with children with disabilities in Vietnam. Their detailed comments helped us revise the questionnaire to produce a fourth and final version.

In April and May of 2008, the assistant lecturers helped respondents complete the questionnaire at the children's community primary schools. To ensure that respondents could understand the items, the lecturers gave each a copy of the questionnaire to read for 15 min, and then talked about the purpose of the research. The lecturers then read each question aloud, checking whether respondents understood (Rural women in Vietnam are very shy and do not speak out when they do not understand or are confused.). Respondents completed the questionnaire themselves and returned it to the lecturers, waiting for a few minutes so that the lecturers could ensure all questions had been answered. The lecturers collected feedback from the respondents so that we could improve the questionnaire for future studies.

### Measures

#### Mental health

We measured general psychological distress (hereafter, ‘distress’) as a general indicator of mental health. Distress was measured using the Kessler 10-item (K10) (31) measure which taps symptoms of non-specific psychological distress, each scored on a 5-point scale from 1=‘none of the time’ to 5=‘all of the time’. Final summed scores have a possible range of 10–50 with higher scores indicating higher levels of distress. Scores ranged from 14 to 47 in the present sample, with the scale exhibiting a satisfactory degree of internal consistency (Cronbach alpha, hereafter ‘*α*’,=0.76). The K10 is a widely used and validated measure, including in Australia, where Vietnamese ancestry is around 0.7% of population (32) and where Vietnam is among the top five countries of birth (other than Australia).

#### Social capital

##### Community participation

We measured frequency and breadth of community participation, and perceptions about participation. We used the 30-item short-form of the ACPQ (20), which taps 14 types of participation: contact with household members, extended family, friends, neighbours, social contact with workmates, organised community activities, religious observance, adult learning, volunteering, giving money to charity, interest in current affairs, expressing opinions, community activism, and political protest. Each item is answered on a 7-point scale from 1=‘never, or almost never’, to 7=‘always, or almost always’. We computed mean scores for each type of participation, with higher scores indicating more frequent participation.

We also computed a breadth of participation index as described elsewhere (20). Briefly, using a multiple regression analysis in which mean scores for all 14 types of participation were entered simultaneously, controlling for key socio-demographic characteristics (see below) and with distress as the dependent variable, types of participation were removed from the equation one-by-one until only significant predictors of distress remained. These items were then dichotomised by median split (to take account of skew in some of the distributions of scores). A score of 1=‘participator’ was assigned to those scoring at or above the median and 0=‘non-participator’ to those scoring below it. These scores were summed to create an index (M=6.1, Md=6.0, SD=3.2). The index displayed an acceptable level on internal consistency (α=0.74), with higher scores indicating greater breadth of participation.

##### Participation perceptions

We measured perceptions (thoughts and feelings) about seven types of community participation that were independently associated with distress in a previous study (20). These were: contact with household members, extended family, friends and neighbours; organised community activities; religious observance; and interest in local affairs. Respondents indicated whether they considered that they participated too little or too much in these types of participation (‘thoughts’), and whether they enjoyed each of them (‘feelings’). Each response was measured on a 7-point scale from 1=‘definitely disagree’ to 7=‘definitely agree’. Mean scores were calculated (M=4.9, Md=5.0, SD=0.92 for ‘too little’ and M=4.8, Md=5.0, SD=1.1 for ‘enjoying’), each demonstrating acceptable internal consistency (0.63 for ‘too little’ and 0.76 for ‘enjoying’ participation).

#### Elements of personal social cohesion

*Social trust*, which is trust in people in general rather than in known others, was measured using the 12-item short-form of the *Organisational Trust Inventory* as adapted for use in the general population (33, 34). Three 4-item sub-scales tap separate dimensions of trust: believing that most people (i) avoid taking excessive advantage of others; (ii) try to negotiate honestly; and (iii) are reliable. Each item is scored on a 7-point scale from ‘definitely agree’ to ‘definitely disagree’. Final mean scores for each sub-scale, and for the full scale, range between 1 and 7, with higher scores indicating higher levels of trust. Mean scores were calculated with the values of 4.7, 5.1, and 4.4 (Md=5.0, SD=0.7; Md=5.25, SD=1; and Md=4.5, SD=0.7 for ‘take advantage’, ‘negotiate honestly’, and ‘reliable’, respectively). The sub-scales and full scale exhibited a lesser degree of internal consistency than found previously in the Australian studies, with *α*-values ranging from 0.38 to 0.53.

*Generalised reciprocity*, the belief that people tend to help each other out without expecting immediate repayment, was measured using the item from the World Values Survey (35). As used, it was ‘generally speaking, would you say that, most of the time, people try to be helpful, or are they mostly looking out for themselves?’ The item is scored 1=‘people try to be helpful’, 0=‘people are mostly looking out for themselves’, with higher scores meaning greater sense of generalised reciprocity (M=4.9, Md=4.5, SD=1.1). As this is a single-item measure, *α* cannot be calculated.

*Optimism* was measured using the Life Orientation Test (36). It includes six items tapping trait (underlying, rather than situation-specific) optimism and includes three positively and three negatively worded items. Consistent with the trust measure, each optimism item was scored on a 7-point scale from 1=‘definitely disagree’ to 7=‘definitely agree’. Final average scores for the scale range between 1 and 7, with higher scores indicating greater optimism (M=4.9, Md=4.83, SD=0.8). The scale exhibited a lower degree of internal consistency than has been found in Australian samples (37), with *α*= 0.35.

*Sense of belonging* and *tangible support* were measured using two sub-scales of the interpersonal support evaluation list (38). The sub-scales assess the degree to which respondents perceive themselves to be included in a social group (sense of belonging) and have people available to offer practical and material support when needed (tangible support). Each sub-scale contains 10 items scored 1=‘yes’ or 0=‘no’. Total summed scores for each sub-scale range between 0 and 10 with higher scores representing higher levels of belonging (M=6.1, Md=6, SD=1.16) and tangible support (M=6.5, Md=6, SD=1.04). With α= 0.20 for belonging, this sub-scale demonstrated poor internal coherence, while, with α=0.53, tangible support displayed only modest internal coherence.

##### Social cohesion

We defined personal social cohesion, using the measures above, as a combination of respondents’ sense of belonging, generalised reciprocity, social trust, confidence (self-efficacy), and hope for the future (optimism). We also included tangible social support because it is related to personal social capital and mental health among women (34), including mothers with children with disabilities (see introduction).

##### Socio-demographic characteristics

Respondents reported their sex, age, level of education, ethnic origin, responsibility for dependent persons (under 18 and over 60 years), being in paid work at least 6 h per week, living alone, and having a government poverty benefit card. They also provided information about their role in the family (primary income-earner and/or caregiver), the condition of their housing (from good to very poor: brick wall and tiled roof; flat house, storeyed house, or other), housing tenure, location of residence (mountains, coast or rural), and financial problems in the last 12 months (such as being unable to pay the rent, selling something to get money, going without a meal, or being unable to afford warm clothes in winter) as well as ownership of each of the seven basic household appliances (television, bicycle, motorbike, car, home telephone, mobile telephone, and washing machine). We asked respondents about family income and the size of family land. However, they could not answer these questions because most were subsistence farmers who rarely measure their land, or they measure it in an unconventional manner (for example, a metric metre equals about 14 metres among these farmers).

#### Extroversion

We controlled for extroversion in this study because extroverted people tend to be more sociable than introverts and, therefore, may participate more in their communities. Because participation is a core part of social capital, this may mean that extroverts have more social capital than introverts. We measured extroversion using the two extroversion items from a 10-item very brief measure of the ‘Big Five’ personality domains (39).

#### Analytic strategy

We start by presenting descriptive statistics for this sample. Relationships between the independent (‘predictor’) and dependent variables were assessed, in the first instance, by examining zero-order (unadjusted) Pearson product-moment correlations. We then examined partial correlations adjusted for all other variables (including socio-demographic characteristics) derived from multiple regression analyses with distress as the dependent variable. The data met the assumptions for the various statistical tests.

Hierarchical linear regression analyses were employed to evaluate the independent contribution made by each predictor variable to explaining variance in distress. Variables were added in blocks. Blocks 1–3 included the control variables and blocks 4–6 the social capital variables, entered in the order suggested by social capital theory (in which participation is hypothesised to generate greater cohesion). Socio-demographic control variables were entered in block 1, type of children's disability (mental, physical, or multi-disability) in block 2, and extroversion in block 3. Block 4 introduced the first of the predictor variables, frequency of participation, followed by perceptions of participation in block 5, and personal social cohesion in the final block, block 6. Changes in standardised beta values from one block to the next were examined to assess the degree of potential mediation, if any, from block to block in the analysis.

## Results

### Socio-economic and demographic characteristics

Respondents’ ages ranged from 25 to 65 years, with nearly three-quarters (72.1%) aged 25–44 years. One-half (50.6%) lived in the mountains, just over one-third (36.6%) on the delta, and 12.8% on the coast. Most mothers (*N*=119, 69.2%) had children with mental disabilities, one-quarter (*N*=44, 25.6%) with physical disabilities, and a few (*N*=9, 5.2%) with multiple disabilities. More than 90% of respondents were living with spouses. The mothers reported poor educational attainment: only 7% of them had completed high school or higher. Most (*N*=90, 52.3%) had completed lower secondary school, and one-quarter (*N*=42, 24.4%) primary school, while a sizeable minority (*N*=28, 16.3%) were illiterate. Most of the respondents were famers (*N*=153, 89%). A few (*N*=6, 3.5%) were government employees or self-employed (*N*=5, 2.9%), with the remainder (*N*=8, 4.7%) being home-makers. Nearly one-half of respondents were both primary income earners (who make money for family living) and caregivers for other family members, including their children with disabilities. Most of the mothers owned their own home which was in good condition (brick wall, tiled roof) and had at least one of the seven essential household appliances (*N*=141, 82% and *N*=158, 91.9%, respectively). However, nearly one-half (*N*=83, 48.3%) of respondents reported having a government poverty benefit card, much more than the average of 16.1% for the rural population in 2008 (40), and nearly three-quarters reported that they had experienced financial problems during the past 12 months.

### Mental health

#### Distress

Scores for distress in the present sample (Table 1) ranged from 14–47 (M=25.07, Md=25, SD=6.02) with very high morbidity rates: only 3.5% of respondents scored <15 (indicating little or no distress), while 81.4% scored 16–30 (moderate distress), and 15.1% scored >30 (severe distress).

**Table 1:** Mean scores and standard deviations for frequency and perceptions of community participation, social cohesion, and general psychological distress

					95% CI	
					
Items	Min	Max	Mean	SD	Lower	Upper
Community participation (frequency)						
Contact with household members	2.5	7.0	5.94	1.01	5.79	6.1
Contact with extended family	1.0	7.0	3.93	0.98	3.93	3.78
Contact with friends	1.0	7.0	3.54	1.05	3.38	3.67
Contact with neighbour	1.0	6.0	3.38	1.15	3.21	3.55
Social contact with workmates	1.0	7.0	3.41	1.32	3.21	3.61
Adult learning	1.0	5.0	1.60	1.06	1.44	1.76
Religious observance	1.0	6.5	2.50	1.54	2.27	2.73
Organised community activities	1.0	7.0	2.98	1.39	2.77	3.19
Volunteer sector activity	1.0	7.0	1.61	1.23	1.42	1.8
Giving money to charity	1.0	7.0	3.81	1.38	3.60	4.02
Active interest in current affairs	1.0	7.0	2.60	1.57	2.37	2.83
Expressing opinions publicly	1.0	6.0	1.89	1.26	1.7	2.08
Community activism	1.0	7.0	2.38	1.76	2.12	2.65
Political protest	1.0	7.0	1.43	1.14	1.26	1.6
Breadth of participation	1.57	5.47	2.96	.67	2.86	3.06
						
Community participation indices (perceptions)						
Too little time for participation	1.0	6.43	4.88	.92	4.74	5.02
Enjoy participation	1.0	7.0	4.84	1.1	4.68	5.01
						
Social cohesion						
Social trust, total score	1.0	6.43	4.75	.676	4.65	4.85
Negotiate honestly	1.0	7.0	5.08	1.38	4.87	5.29
Don't take advantage	2.83	6.42	5.10	1.03	4.95	5.23
Keep commitments	1.0	7.0	4.45	1.12	4.28	4.62
World Values Survey trust	1.50	7.0	4.49	1.17	4.31	4.66
World Values Survey reciprocity	2.0	6.75	4.92	1.14	4.75	5.09
Optimism	1.5	7.0	4.93	.84	4.81	5.06
Sense of belonging	2.0	10.0	6.42	1.57	6.18	6.66
Tangible support	2.0	10.0	6.81	1.92	6.52	7.10
General psychological distress	14	47	25.07	6.02	24.16	25.98

**Fig. 1:**
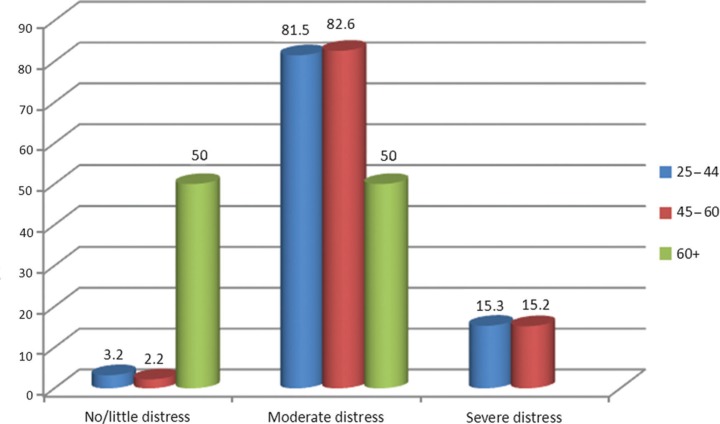
Proportions of mothers reporting no/little, moderate, and severe distress by age group.

There are no Vietnamese data with which to compare these findings, but Australian norms are 68%, 29%, and 3% (Kessler et al., 2002). Younger mothers reported the greatest distress (Fig. 1).

### Social capital

#### Community participation

Most mothers participated infrequently in community activities (Table 1). The most common forms of participation were contact with household members, with extended family, with friends, with workmates, and giving money for charity; they undertook less community activism, adult learning, volunteer activities, expressing opinion, and political protest. Most respondents reported that they spent too little time in community participation but that they enjoyed these activities when they could. In a multiple linear regression analysis, 9 of the 14 types of participation were independently associated with distress, 3 of them with less distress (in the order: neighbours, adult learning, and friends) and 6 with greater distress (activism, extended family, immediate household, political protest, workmates, and religious observance). To assess any impact of breadth of participation, two indices were constructed by summing the items endorsed. One measured breadth of participation across types of activities that were associated with less distress and one with more.

#### Personal social cohesion

Mothers reported moderate to high levels of all components of personal social cohesion (trust, reciprocity, optimism, sense of belonging, and tangible support). However, the components of social capital were only modestly associated with mothers’ distress (Table 2). Frequency of participation, trust, and sense of belonging were significantly negatively related to mothers’ distress such that greater participation, trust, and sense of belonging were associated with less distress. The components of social capital were positively correlated, as expected, such that those scoring higher on one element were likely also to score higher on another.

**Table 2:** Pearson Product Moment correlations among social capital variables and mothers’ general psychological distress (K10 scores)

	2	3	4	5	6	7	8	9	10	11	12	13	Distress
1. Breadth (less distress)	0.62***	−0.07	0.03	−0.04	−0.12	−0.05	0.08	0.09	0.08	−0.36***	0.00	−0.08	−.14
2. Breadth (more distress)		−0.06	−0.07	−0.03	−0.21**	0.06	0.09	−0.10	−0.01	−0.29***	0.05	−0.13	0.21**
3. Too little participation			0.41***	0.32***	0.40***	−0.01	0.21**	0.22***	0.42***	0.06	−0.18*	0.00	0.04
4. Enjoy participation				0.10	0.22**	−0.12	0.09	0.10	0.32***	−0.07	−0.16*	0.08	−0.17*
5. Social trust (full scale)					0.65***	0.47***	0.76***	0.17*	0.29***	−0.02	−0.11	0.18*	0.02
6. Negotiate honestly						−0.13	0.35***	0.26***	0.38***	0.11	−0.12	0.11	−0.02
7. Do not take advantage							0.04	−0.06	−0.06	−0.03	−0.12	0.20**	0.00
8. Keep commitments								0.12	0.22**	−0.12	0.01	0.04	0.06
9. WVS trust									0.21**	0.08	−0.17*	0.15	−0.24**
10. WVS reciprocity										−0.06	−0.15	0.19*	−0.02
11. Sense of belonging											−0.01	−0.06	0.00
12. Tangible support												−0.34***	0.07
13. Optimism												–	−0.21**

* *p*-value < 0.05

** *p*-value < 0.01

*** *p*-value < 0.001.

Hierarchical multiple linear regression analyses were used to assess the relative contributions of the different measures in explaining the variance of mothers’ psychological distress (Table 3). In step 1, ‘living in a mountainous area’ (less distress) and having the role of combined carer and income earner (more distress), each made significant independent contributions to explaining variance in mothers’ distress. Children's type of disability made no significant contribution to the model in step 2. Extroversion (specifically, being ‘reserved’) made a small contribution to explaining mothers’ (lower) distress when added in step 3, and also resulted in the role of ‘combined carer and income earner’ becoming non-significant. This suggests that being reserved accounted for the relationship between this role and being more distress.

**Table 3:** Multiple hierarchical linear regression analysis showing blocks of predictors of mothers’ general psychological distress (K10 scores)

	B	SE B	Beta	R^2^ change	Adj. R^2^
1. Socio-demographic characteristics					
Mountainous area	−2.63	0.92	−0.22**	0.08***	0.08***
(Combined earner and carer)##	1.92	0.92	0.16*		
2. Child's disability				*ns*	*ns*
3. Extroversion					
Mountainous area	−3.18	0.87	−0.27***	0.04**	0.09***
Being ‘reserved’	−0.69	0.27	−0.19**		
4. Frequency of participation					
Mountainous area	−3.72	0.83	−0.31***	0.21***	0.27***
Being ‘reserved’	−0.60	0.26	−0.16*		
Immediate household	1.72	0.88	0.14*		
Extended family	1.06	0.51	0.17*		
Friends	−1.37	0.50	−0.24**		
Neighbours	−1.28	0.41	−0.24**		
Workmates	1.00	0.34	0.22**		
Adult learning	−2.77	1.08	−0.20**		
Activism	3.16	0.91	0.26**		
Political protest	2.68	1.20	0.18*		
5. Breadth of participation				*ns*	*ns*
6. Perceptions of participation				*ns*	*ns*
7. Social cohesion					
Mountainous area	−3.45	0.83	−0.29***	0.02*	0.29***
Being ‘reserved’	−0.59	0.26	−0.16*		
Immediate household	1.80	0.87	0.15*		
Extended family	1.07	0.50	0.18*		
Friends	−1.34	0.49	−0.23**		
Neighbours	−1.14	0.41	−0.22**		
Workmates	0.85	0.35	0.19*		
Adult learning	−2.72	1.06	−0.20**		
Activism	3.03	0.90	0.25***		
Political protest	2.49	1.19	0.17*		
World Values Survey trust	−1.78	0.83	−0.15*		

## *Note*: Predictor shows mediation effect later in the model (displayed in brackets).

* *p*-value<.05

** *p*-value<.01

*** *p*-value<.001; ‘*ns*’ means not significant.

In step 4, frequency of participation variables were entered with 8 of the 14 types of participation making a significant and, together, large (21%) independent contribution to explaining variance in mothers’ distress. Three of them contributed to less distress (in the order, friends, neighbours, and adult learning) and five to greater distress (activism, workmates, political protest, extended family, and immediate household). Breadth of participation in step 5 and perceptions about participation in step 6 did not contribute explained variance to the model. In the final step, one component of social cohesion (believing people could ‘be trusted’) made a further, small contribution to explaining variance in mothers’ distress, bringing total adjusted variance explained to 29%. In the final model, less distress was predicted by, in the order, living in a mountainous area, seeing friends and neighbours, participating in adult learning, and trusting others. Greater distress was predicted, in the order, by engagement with activism, workmates, extended family, political protest, and immediate household.

## Discussion

In Vietnam, as elsewhere, mothers with children with disabilities have very high levels of distress. Younger mothers in this sample reported the greatest distress, consistent with international findings that mental health problems are more prevalent among younger than older people. In his review (41), Bailey noted that many different tools have been used to measure mental health in different studies, complicating the comparison of findings across studies. The present study was the first to translate and use the K10 measure of general psychological distress. Compared with prevalence studies focused on *depression*, we found relatively high rates of *distress* among this sample of mothers. This is not surprising, as distress is a more-encompassing concept than depression.

Although the mothers in this study reported high levels of social cohesion, except for one measure of trust, components of cohesion were not linked to their mental health, unlike in developed economies. As in other studies (2, 3, 26, 41–43), these mothers had very low levels of community participation. Participation was quite strongly linked to distress noting that, among these mothers, except for three types of activity (neighbours, friends, and adult learning), participation was linked to *greater*, and not to *less*, distress. This finding has been observed internationally in developed countries (44), and among Aboriginal Australians (20, 22, 23) who, while living in a developed economy, tend not to share in its benefits. It is possible that, being part of a severely marginalised group (a circumstance that is much more prevalent among women than among men), participation is more difficult and more costly, in many ways, to these chronically stressed and under-resourced parents (22).

We found other differences between these exceptionally pressured, low-income country mothers compared to parents of children with disabilities in other countries. For example, socio-demographic characteristics and the nature of the child's disability did not help explain variance in mothers’ distress, and neither did most of the components of social cohesion. That said, social capital (and particularly – at the individual level – social cohesion) has been found to be helpful for common mental disorders among young mothers in developing countries, including Vietnam (45). However, the mothers in this earlier study were not parents of children with disabilities. The present findings suggest, perhaps, that their circumstances are so unusual or extreme that a wholly different explanatory framework needs to be developed. For example, stigma and misunderstanding may be significant factors (46), as may the quality of relationships (47). To better understand the circumstances of these mothers, future studies would benefit from the inclusion of a comparison control group of mothers whose children do not have disabilities where the study is conducted in an otherwise identical setting.

The issue of being under-resourced and marginalised is enormously important. For example, it has been reported that families with children who need to be assisted by technology receive much less help with childcare (day care, babysitting, help from relatives, and professional nursing) than other families (48), though they need help so much more. Being isolated may also contribute to mothers’ distress: mothers who provide full-time childcare for children with disabilities report greater depression than mothers who do not (49).

There are some substantial weaknesses in the present study, key among them the understandably small sample size (which makes it difficult to be confident in the statistical estimates and impossible to conduct certain statistical tests) and the use of measures that have been translated and deployed for the first time in Vietnam (making it difficult to be sure that the correct concept is being tested, and tested correctly). Nevertheless, mothers could and did complete the survey, and it was meaningful to them. The sample was appropriately selected, with a strong response rate, and represents an important and frequently overlooked group in Vietnamese society. The study provides insights into the complicated relationship that mothers of children with disabilities have with their communities, and the many ways in which engagement can be difficult, possibly even harmful for their mental health.

## Conclusion

Mothers of children with disabilities in rural Vietnam are very distressed and social capital, especially the participation component, significantly predicts this distress. Building social capital among these mothers could be helpful for their mental health but, if this strategy is used, it will be essential to ensure that women are involved in types of participation that are associated with *better* mental health (friends, neighbours, learning) and not encouraged to participate in those that are not. This may be especially important in a context in which financial support is not easily available or, possibly, not the most helpful kind of support (11), wherein social capital may appear a preferable alternative. Opportunities for adult education may be an alternative strategy, as this seems to be a positive experience for these highly disadvantaged mothers.
